# M-GRCT: A Dynamic Circular Economy Model for the Optimal Design of Waste Management Systems in Low-Income Municipalities

**DOI:** 10.3390/ijerph19052681

**Published:** 2022-02-25

**Authors:** Camilo-A. Vargas-Terranova, Javier Rodrigo-Ilarri, María-Elena Rodrigo-Clavero, Miguel-A. Rozo-Arango

**Affiliations:** 1 Programa de Ingeniería Ambiental y Sanitaria, Universidad de La Salle, Carrera 2 # 10-70, Piso 6-Bloque A, Bogotá 111711, Colombia; cvterranova@unisalle.edu.co; 2 Instituto de Ingeniería del Agua y Medio Ambiente (IIAMA), Universitat Politècnica de València, 46022 Valencia, Spain; marodcla@upv.es; 3 Quipus Consultores SAS, Carrera 8 # 16-36, Cota 250010, Colombia; mrozo09@unisalle.edu.co

**Keywords:** circular economy, solid waste management, recycling, environmental management

## Abstract

This article introduces M-GRCT, a circular economy decision support model for the design of recyclable waste management systems in low-income municipalities. The model allows for performing calculations on a set of two scenarios integrating a sociocultural dynamics assessment, this being a characteristic feature of this type of municipalities. The model also integrates the analysis of the remaining variables usually addressed in solid waste management schemes while considering topics such as reduction of the carbon footprint due to activities such as the transport of recyclable waste, the generation of leachates, the generation of greenhouse gases and the promotion of an increase in the number of associated recyclers and selective routes. The economic evaluation of the different implementation scenarios is supported by a dynamic tool called DATA4 (a macro-type array accompanied by two control panels programmed in Visual Basic and dashboards by Power BI). M-GRCT constitutes a tool for the promotion of good environmental practices and the identification of strategies for the promotion of local development mechanisms. Results provided by the model contrast with those obtained by traditional linear economy approaches. An illustrative example of the application of the M-GRCT model is shown. The model was used to simulate the municipal solid waste managing system of the municipality of Guateque (Colombia). The results show the importance of integrating both economic and environmental costs to optimally allocate governmental and private resources when the recycling rate is expected to increase in the next 10 years.

## 1. Introduction and Objectives

The increasing demand for natural resources, caused by population growth, induces a growing tendency toward promoting an efficient use of the available resources [1,2]. This causes many to criticize the linear economy, since it only seeks economic growth without taking into account social and environmental impacts [3,4,5]. The circular economy emerges as an alternative to the current linear economy [6,7,8], with the aim of extending the useful life of products and the components and materials in circulation, without loss of value, while minimizing final rejection of waste [9,10].

The importance of implementing a circular economy strategy is also highlighted by its having been declared one of the key solutions to help meet the objectives of the Paris Agreement [11]. It is also considered a key strategy to achieve the EU climate goals [12] and the Sustainable Development Goals [13,14]. It could even help increase gross domestic product, improve employment opportunities and reduce greenhouse gas (GHG) emissions [15].

However, there are also limitations in the application of circular economy models [16]. Currently, the circular economy presents a technologically and economically profitable vision of continuous growth in a world with scarce resources [17], without considering the social and political implications [18] and without exhaustively analyzing the necessary social and institutional conditions [17,19]. Another important limitation is the existence of a bias of developed countries towards the circular economy, allowing continuous economic, industrial and global growth without reflecting the impacts on developing countries [18]. Only very recent approaches have focused on incorporating the social dimension in circular economy models [16,20].

Solid waste management plays a fundamental role in the implementation of this new economy, since the circular economy is an economic mechanism whose main objective is to eradicate waste and ensure an optimal use of resources. To build this closed-loop structure, the key tasks in the circular economy are reuse, restore, remanufacture and recycle. In this way, it is intended to reduce the input and waste of resources and, as a consequence, pollution [21,22]. Applying circular economy approaches in waste management systems provides the following benefits [23]: (i) provision of affordable waste collection services to all income areas, (ii) increase in the amount of waste collected and recycled, (iii) health improvement at household levels, (iv) reduction in GHG emissions, (v) direct and indirect job creation and (vi) increase in the application of compost to improve agricultural soil fertility.

In recent years, several attempts have been made to implement the principles of the circular economy in the comprehensive management of urban solid waste [24,25,26,27,28,29,30], following the guidelines of the European regulations [31]. Some of these attempts are based on applying mathematical models that allow for optimization and improvement of waste treatment, minimizing disposal in landfills and promoting waste recirculation and recycling. Waste has been considered another resource. For example, some authors proposed a sequential optimization model that allows for knowing the roles that municipalities and recycling companies play, respectively, in improving the classification, collection and reuse of waste, considering the economic impact [32]. Other research analyzed an Egyptian waste management system and its potential for prevention and recovery, contributing to highlighting the best collection method that leads to the lowest cost and maximum benefit for all interested parties in waste management systems in Egypt by considering direct and indirect benefits to achieve sustainability [33]. It is also sought that the sustainable management of waste be profitable [34], through the application of an economic model that optimizes waste management by directing it to “zero discharge”. 

Recent studies show the importance of implementing circular economy techniques in rural areas [35,36,37,38]. The objective of this work is to present a new dynamic circular economy model, called M-GRCT, which is applicable to low-income municipalities and which allows for analyzing the benefits and drawbacks of the circular economy approach compared to the linear economy approach. The sections of this paper are organized as follows:

Section 2, “Materials and Methods”, describes the main characteristics of low-income municipalities (Section 2.1) which have been taken into account in the design of the M-GRCT numerical model (Section 2.2). The detailed description of the model is provided in Section 2.3, Section 2.4, Section 2.5, Section 2.6 and Section 2.7. Section 3, “Case study”, shows an application of the model to a low-income municipality in Colombia. Section 4 and Section 5 include the discussion of the results, the main conclusions and future research lines.

## 2. Materials and Methods

### 2.1. Low-Income Municipalities: General Characteristics

In Colombia, there is a national law (Number 617) establishing/categorizing municipalities and setting the annual current income in accordance with this. Article 6 of the law sets specific amounts of annual money delivered by the central government [39]. This categorization allows for certain kinds of administrative functions such as investment access, improvement of public management and the allocation and distribution of national transfers [40]. To be classified inside the fifth category, a municipality must have a population between 10,001 and 20,000 inhabitants or show a current income between 15,000 and 25,000 times the monthly legal minimum wage (EUR 228 in January 2022) [41].

The M-GRCT model was developed to be used on low-income (fifth and sixth category) municipalities whose annual average incomes do not exceed EUR 53,000,000 per year. Other characteristics of these municipalities include (i) a population lower than 20,000 inhabitants and (ii) MSW per capita production lower than 0.70 kg/person-day [42]. Usually, these municipalities are located far from large urban centers, where socioeconomic vulnerability is accentuated and the lack of coverage in the provision of public services is notorious, especially those services related to drinking water, sewerage and cleaning [43].

This lack of coverage on essential public services is essentially due to the lack of political-administrative management by the competent authorities, who in principle should be in charge of them. Not guaranteeing the provision of services not only violates the collective rights of these populations but also affects their socio-economic development. In these areas, there is a significant lack of job offerings and private investment is low [44].

Specifically, in relation to the public cleaning service, the provision of the service may not be available or may lack technical and administrative capacity to provide comprehensive management of solid waste. This induces the disposal of municipal wastes in uncontrolled landfills/dumps or even their incineration as open burns. Public health problems and negative environmental impacts are generally produced due to the proliferation of pests, the generation and non-treatment of leachate and the emission of greenhouse gases [45].

### 2.2. M-GRCT: A Numerical Model for the Simulation of Recyclable Solid Waste Management Systems

M-GRCT is a numerical model for the simulation of recyclable solid waste management systems based on a circular economy scheme. The model provides a decision-making tool for the municipalities under study, allowing them to study the implementation of recyclable waste management strategies when applying a circular economy. This is done by calculating financial indicators that measure the economic viability of the commercialization of this type of waste.

**Figure 1:**
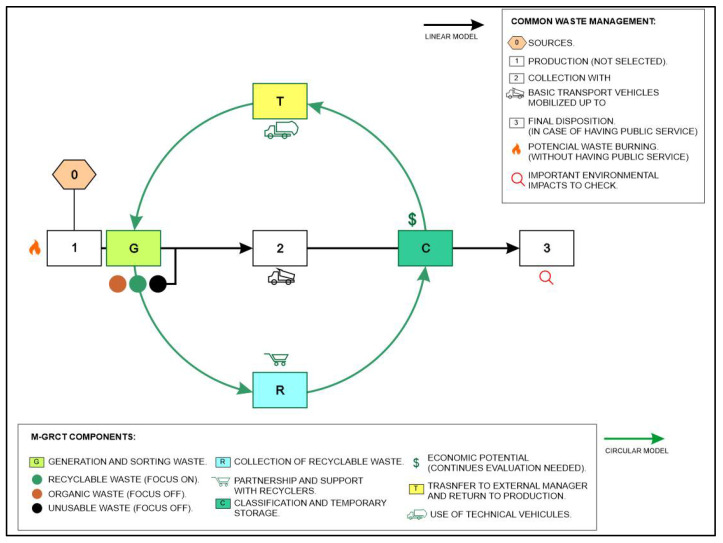
Conceptual scheme of the M-GRCT model.

The model was designed to analyze the following four components of the waste management system: (i) recyclable solid waste generation and segregation (G), (ii) recyclable waste collection (R), (iii) classification and temporary storage in collection centers (C) and (iv) transfer to external managers and return to the production chain (T). Figure 1 shows the conceptual scheme of the M-GRCT model.

As shown in Figure 1, waste management systems must consider the extraction of raw materials as a Stage 0 for the production of goods and services. This initial stage also implies the generation of waste. Afterwards, if a conventional linear system is available at the municipality, waste is usually collected by the public cleaning service using non-technical vehicles and is finally disposed (eventually after some kind of treatment) on a sanitary landfill. 

By contrast, the proposed circular model considers the segregation of the main types of waste. Non-usable and organic wastes are not considered by M-GRCT, which only considers recyclable waste as a target. The circular model fundament is strongly based on the alliance and active participation of waste pickers who potentially carry out the recycling activities in economically depressed municipalities with few job opportunities. In addition, the evaluation of the actual economic and marketable possibilities of recyclable waste is needed. Waste sorting and storing activities can be performed in temporary collection centers that may or may not exist in these municipalities. Finally, the recycled products are returned to the production chains.

Other elements identified in the model’s conceptual scheme include the impacts generated by the final disposal of waste if a public cleaning service supported by technical vehicles is not available. In such a case, uncontrolled burning of waste is still being used in these municipalities, inducing important environmental and sanitary problems.

The implementation of a circular approach such as the one considered by the M-GRCT model allows for avoiding the generation of significant negative environmental or economic impacts which are associated with waste management models based on linear economy schemes, in which waste is finally disposed in sanitary landfills and further potential use alternatives are discarded. M-GRCT considers the existence of economic incomes related to (i) specific taxes or rates imposed over the collection and transport of recyclable waste or (ii) the commercialization of recyclable products by external managers. Additionally, the model considers the existence of supervision activities on every managing stage which continuously promotes the system’s improvement.

Based on all the available information, M-GRCT allows for simulating different scenarios to optimally design the waste management system in economically depressed municipalities. The four model components are described below. 

#### 2.2.1. Recyclable Solid Waste Generation and Segregation (G)

**Figure 2:**
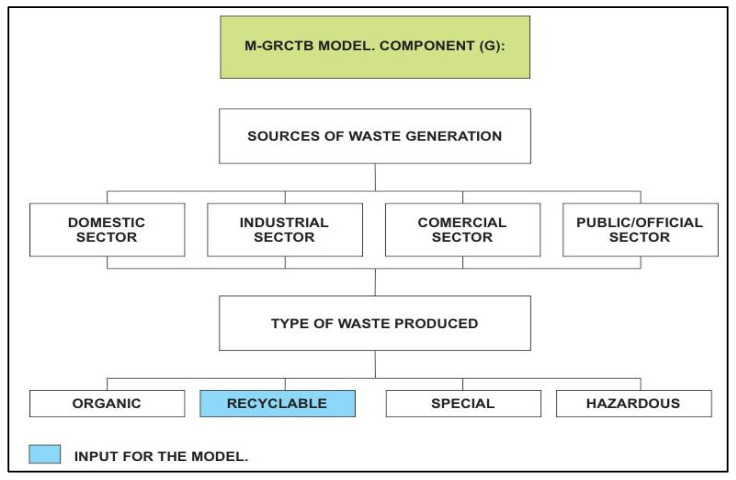
Recyclable waste generation sources considered by the M-GRCT model: Component (G).

The M-GRCT’s first component (G) determines the sources of generation of recyclable solid waste for each municipality that can be valued with the model. These sources usually correspond to the domestic, commercial, industrial and official sectors, as shown in Figure 2. 

In each of these recyclable waste generation sources, it is essential to apply segregation techniques, that is, the specific selection and separation of each recyclable waste type. If this segregation is carried out in practice, the recycling of waste is highly facilitated [46].

**Figure 3:**
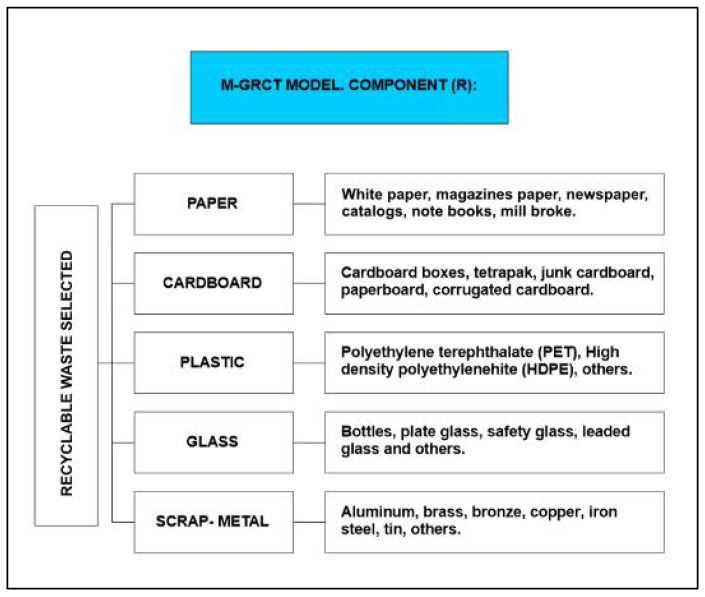
Recyclable waste classification considered by the M-GRCT model: Component (R).

Recyclable waste is usually classified in five categories: paper, cardboard, plastic, glass and scrap-metal, with each one being subdivided into subcategories, as shown in Figure 3. This information is used to compile data on the annual production of recyclable waste while allowing for estimating the reduction of recyclable waste disposed of in sanitary landfills and the economic benefits derived from their possible commercialization.

#### 2.2.2. Recyclable Waste Collection (R)

The M-GRCT’s second component (R) describes the recyclable waste collection system and carries out a census of recyclers classified by waste category. These recyclers are those who actually carry out the waste collection in low-income municipalities. In addition, based on the recyclable waste categories shown in Figure 3, it is important that citizen initiatives be encouraged through environmental education, so that the collection of solid waste is promoted. As explained above, if waste is stored in favorable conditions, it can be collected efficiently [47].

Additionally, from a technical perspective, it is important that selective collection routes be defined and that correct waste separation at the source—especially with recyclable waste—be implemented. These actions ensure that the implementation of circular economy models and the application of waste treatment technologies are optimal and generate satisfactory results [48], so that potentially usable residues are not discarded as raw material.

For these reasons, it is important to implement circular economy models for professional waste pickers or waste picker associations in low-income municipalities. In low-income municipalities it has been seen that waste picker income is not sufficient to allow them to carry out their activity permanently. Larger unions with greater administrative control usually find private recycling companies that dominate and control the market [49].

#### 2.2.3. Classification and Temporary Storage in Collection Centers (C)

**Figure 4:**
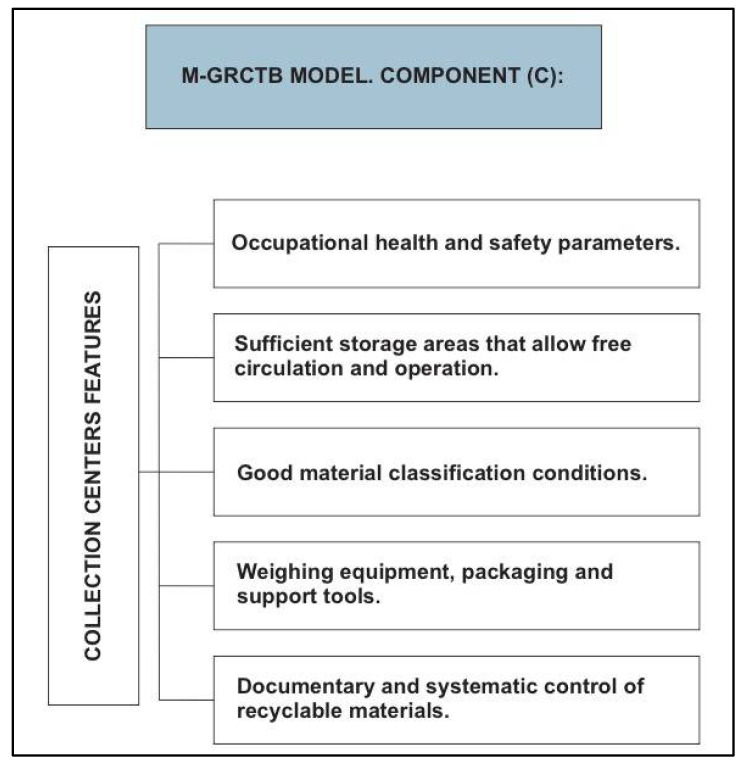
Logistical and administrative characteristics of a waste collection center. M-GRCT model: Component (C).

The M-GRCT’s third component (C) performs a characterization of the collection centers where recyclable waste is classified. The center’s main technical features, dimensions and administrative organization are detailed as shown in Figure 4. Based on this information, the M-CGRT model classifies the collection centers and solid waste managing infrastructure.

Within the collection centers, the reception, weighing, classification and recyclable waste storage processes are performed in a systematic and comprehensive manner. These activities are carried out through manual, mechanical or mixed operation systems by environmental reclaimers who have as their main purpose the performance of a preliminary treatment to the waste in order to reduce the final amount disposed of in a sanitary landfill [50].

#### 2.2.4. Transfer to External Managers and Return to the Production Chain (T)

The M-GRCT’s fourth component (T) considers the closure of the circular economy cycle. Recyclable waste is transferred to external managers, allowing for and promoting the extension of the useful life of the byproducts obtained from waste and thereby increasing its amount and utility [51].

For this reason, strategies such as reuse or eco-design allow recyclable waste to be kept longer in the production chain due to its potential for use, as well as its durability, generating an increase in the waste value and the chance for it to be reintroduced in different production lines as raw material [52].

The circular economy is characterized by three different research levels depending on the scale of the analysis [53]. At a municipal level, the circular economy is highly focused on the development of eco-cities or eco-municipalities through the development of environmental policies and administrative participation of governments. That is the reason why implementing circular economy strategies in low-income municipalities increases the likelihood that business models will improve their local economy and induce benefits in terms of the economic income of the local businesses and small companies at the medium term.

### 2.3. DATA4: The M-GRCT Computer Support Tool 

**Figure 5:**
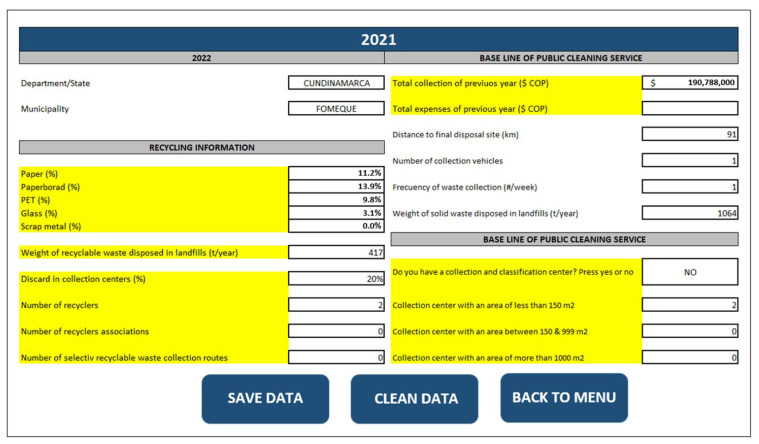
General view of the DATA4 tool’s main menu.

M-GRCT was implemented in a computer tool called DATA4 which allows for developing the economic comparison between the implementation of a solid waste management model based on the linear economy and the establishment of a model applying the circular economy. DATA4 was built in Excel^®^ enabling macros with absolute references to facilitate user interaction with its interface [54]. DATA4 takes into account the following technical-operational factors of municipal solid waste management systems: (i) operating costs, (ii) collection costs, (iii) transportation costs to the final disposal site, (iv) final disposal costs, (v) infrastructure costs, (vi) socioeconomic characterization of the municipality, and (vii) volume of recyclable waste. Figure 5 shows a general view of the DATA4 tool’s main menu.

All the information is entered in a second sheet that contains a record with the following data: (i) department, (ii) municipality, (iii) category, (iv) public service provider company, (v) number of recycling stations by area range, (vi) annual recyclable waste and (vii) financial information of the cleaning service company (total collection for the previous year, expenses, administrative payroll, workers payroll, staffing, cost of collection and transportation to final disposal and cost of final disposal). 

The financial information is an input for the economic valuation of the waste management process currently developed. Based on this information, the cost-benefit analysis of the model application is subsequently carried out [55,56,57]. A Visual Basic^®^ programmed macro processes all this information, which is stored in a specific place (third block of the DATA4 tool). This allows for the classification into scenarios through a conditional that describes the existing infrastructure by municipality and the type of waste collector system.

DATA 4 allows the user to analyze two predefined scenarios which were designed to represent the recyclable waste management system of a low-income municipality. These scenarios were designed considering the actual availability of recyclable waste facilities in the municipality.

- Scenario 1 corresponds to municipalities that do not have physical infrastructure and machinery to perform any recycling operations that are classified as large or medium collector of recyclable waste. Actual considerations about the construction and operation of waste treatment infrastructure depend on the municipal budget and the municipality’s characteristics. Specifically, the definition of the dimensions of the waste treatment infrastructure is directly related to the population, the generation of waste and the municipal budget [58]. Two predetermined areas were considered: collection centers of 200 and 350 m^2^. To estimate the economic costs of the work, existing guidelines [58] were taken as a basis.
- Scenario 2 corresponds to municipalities with existing recycling centers equipped with machinery and considered by DATA4 as great collectors. The improvement of these infrastructures refers to the installation of rigid floors, odor emission control systems, fire prevention and control systems, separation of areas for waste reception activities, such as weighing, selection and classification, and temporary storage of recyclable materials and rejection materials.

Predefined recyclable waste classification ranges were included as follows: small collectors (0–1020 t/year), medium collectors (1021–3100 t/year) and high collectors (>3101 t/year). These ranges were defined by applying the Jenks algorithm (natural breaks method by Arcgis^®^) [59] in the sample of the volume of recyclable waste stored in sanitary landfills in locations with similar socioeconomic situations as the low-income municipalities under study.

DATA4 automatically exports the results obtained in a compatible way to the format of the data developed in the macros through Power BI^®^. Afterwards, the relationships between the variables are visualized through graphs to unify and synthesize all this information. 

**Figure 6:**
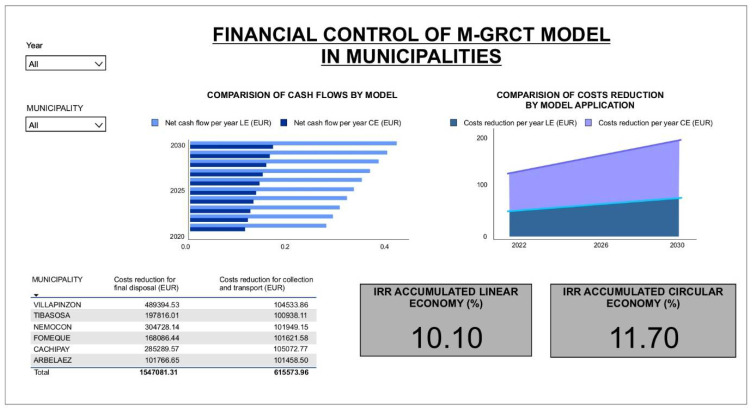

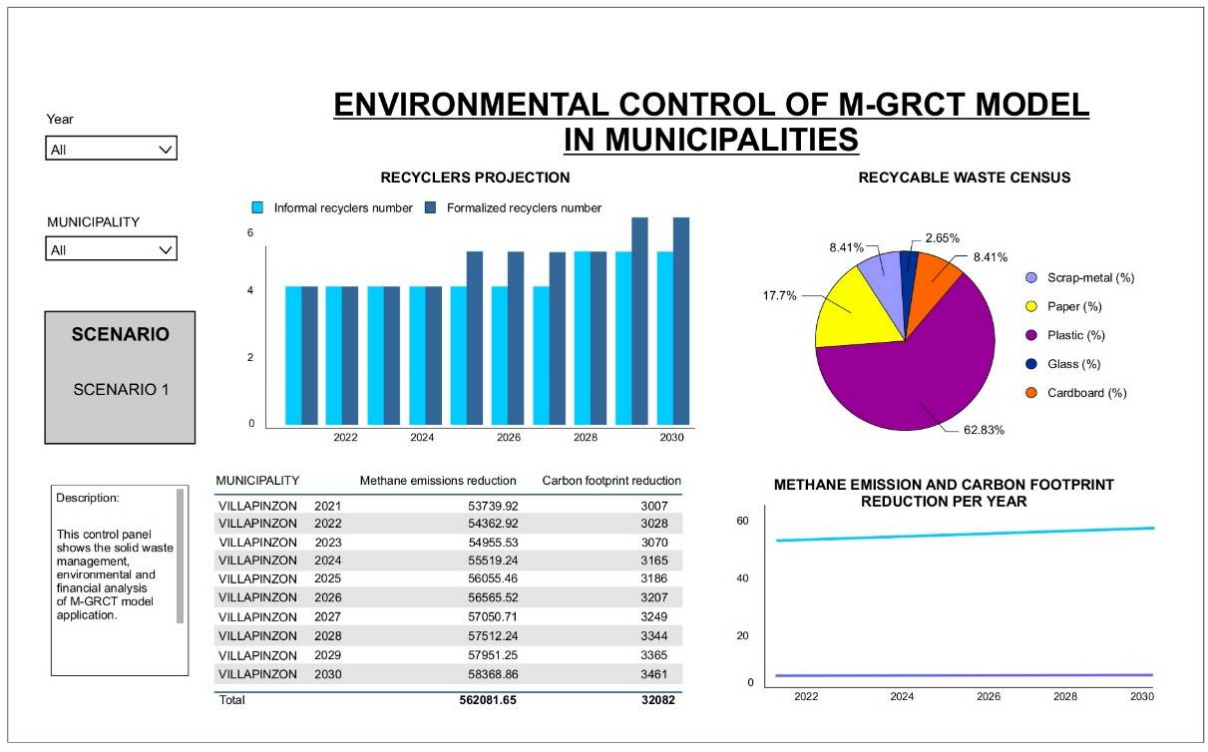
DATA4 dashboard.

Figure 6 shows an example of the two windows of the DATA4 dashboard (Power BI^®^) designed to obtain visual results.

The graphical interface of DATA4 allows for visualizing the results obtained when the model runs. In particular, a set of financial trends, the composition of recyclable waste, the carbon footprint reduction trend and the projection of recyclers joining the process allows the user to identify more strongly the advantages of the M-GRCT model. All the visible variables in the dashboard are compared with the traditional linear model, allowing a practical analysis to promote decision-making to mean the implementation of the model in low-income municipalities. The dashboard is automatically updated with the data entry in the main menu of DATA4.

### 2.4. Definition of Scenarios-Based Recyclable Waste Management Criteria

Simulation scenarios are considered as the context in which the M-GRCT model could be implemented in low-income municipalities [60]. In other words, the definition of simulation scenarios requires establishing the conditions that guarantee its applicability. These conditions depend on the recyclable waste management criteria recorded in Components (G) and (R). The following model projections based in these two previous stored components are obtained by M-GRCT: (i) the existence, use and characteristics of infrastructure, (ii) the recyclable waste collected and stored in final disposal sites and (iii) the availability of machinery for recycling activities. Table 1 shows the DATA4 tool’s complete list of parameters.

### 2.5. Macro-Type Array Processing

Within the matrix considered in the macro sheet the model evaluates two large modules: an environmental context and a financial context. This must be achieved within the implementation of the circular model. Each of the two modules includes a set of features that infer arithmetic processes that are performed by the interface. 

The summary of these features and related mathematical processes is shown in Table 2 and Table 3.

### 2.6. Running the M-GRCT Model Using DATA4 

M-GRCT Component (G) is integrated into Block 2 of the DATA4 tool to record the characterization of recyclable waste. The annual production (t/year) of each type of recyclable waste is requested (Table 4). The objective of the model is to estimate the increase in the annual generation of recyclable waste for each simulation scenario.

The weight of recyclable waste per year that is produced for final disposal (t/year) is incorporated as Component (R) in the model (Table 5). These values are stored in the information record block of DATA4. Component (R) is used by the macro to classify municipalities according to the collector type mentioned in Block 3 of Table 1. In the same way, the monitoring control panel performs a multi-year comparison between the collection of recyclable waste (t), the number of recyclers and the number of selective routes after the execution of different scenarios for the reference year.

A potential categorization of the municipality (Table 6) is essential to determine the collection and temporary storage center dimensions (C), since it is directly related to the population, the waste generation ratio and the municipal budget.

Afterwards, the simulation scenarios must be defined (as established in Section 3.4). DATA4 develops an economic assessment of the implementation of each scenario, considering a 10-year operation period of a collection center for recyclable waste and assessing the economic viability through financial indicators such as the internal rate of return (IRR), the net present value (NPV) and the return investment period (RIP). 

A conditioning factor for the evaluation using NPV is performing a basic cash flow (ratio of annual income and expenses) since, if the expenses are greater than the incomes, NPV will be negative. In such a case, the financial evaluation is estimated only using the cash flow projection.

The return of waste to the production cycle (T) is a variable considered in the control panel that shows the results of the economic evaluation. This dashboard includes the following indicators: cost reduction due to the implementation of scenarios, IRR for the waste management linear and circular models and NPV for the waste management linear and circular model. These results are dependent and proportional to the annual refund percentage (which is progressively increased by DATA4 from 1% to 10%) and the sales projection of each type of recyclable waste (Table 7).

### 2.7. Financial Viability of the Circular Economy Model

The financial viability of a circular economy model is represented by financial indicators such as the NPV, the internal rate of opportunity (IRO), the IRR and the period of return or payback. NPV is related to the difference between the capital investment in a project and its expenditures; therefore, projects with a positive NPV will be more economically feasible compared to projects with a negative NPV [56]. In the same way, a project is more profitable in economic terms if its IRR is greater than the opportunity cost of capital (IRO), because investment opportunities in which the opportunity cost is lower than the rates of return guarantee higher profits. Regarding the payback period, economic theory indicates that projects in which the invested capital is recovered in less time increase the possibility of financial success [61].

Regarding the results of the economic valuation of the linear and circular solid waste management models considered in this work, they are analyzed through a cost-benefit balance and the valuation of the aforementioned financial indicators. The results are shown in DATA4 control dashboard. They depend on the recycling rate, which is progressively increased automatically by DATA4 taking into account a trend analysis of the municipalities under study.

The control dashboard shows the comparison (bar graph) between the value of total income and expenses of waste collection for the linear model and those estimated for every simulated scenario of the circular model. In addition, the control dashboard indicates (using a linear graph) the cost reduction for the implementation of a specific scenario, its economic viability analyzed according to the IRR and the investment return period for its implementation.

The monitoring control dashboard makes a multi-year comparison between the recyclable waste finally stored in the sanitary landfill after the execution of any of the scenarios and the corresponding values for the base year. This comparison is made using bar diagrams. In the same way, the control dashboard shows the annual waste amount that enters the collection center by type of waste (paper, cardboard, plastic, glass and scrap), its percentage in a circular diagram and the economic income obtained by its sales in a 2D horizontal bar chart.

The whole simulation using M-GRCT allows for obtaining a financial evaluation of the waste management system according to the principles of the circular economy: linking new production strategies to the waste cleaning service, ensuring economic and environmental sustainability and offering a sufficiently long useful life [62].

## 3. Case Study: Using the M-GRCT Model on the Municipality of Guateque (Colombia)

### 3.1. Description of the Study Area and Scenario Selection

**Figure 7:**
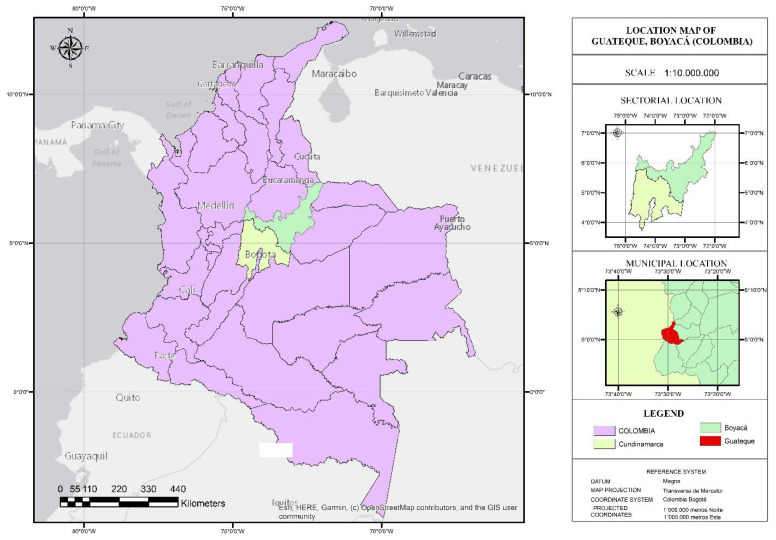
Geographic location of the Guateque municipality.

In order to demonstrate the applicability of the model in a specific case study, the M-GRCT was used to analyze the waste management system of the municipality of Guateque, located in the department of Boyacá (Figure 7). It has an approximate area of 36.04 km^2^ and, according to official data for the year 2020, a population of 10,904 inhabitants [63]. From an economic perspective, Guateque is not a municipality that shows significant economic growth, as a result of the high dependence of the economy on agriculture without further technological development, the virtual non-existence of an industrial sector and the presence of a weak commercial and services sector in which there are no employment opportunities to retain the population that tend to migrate to bigger cities [64].

Table 8 shows the municipality registry, reporting general information of the cleaning service, including location, category, baseline of the public cleaning service and technical details.

**Figure 8:**
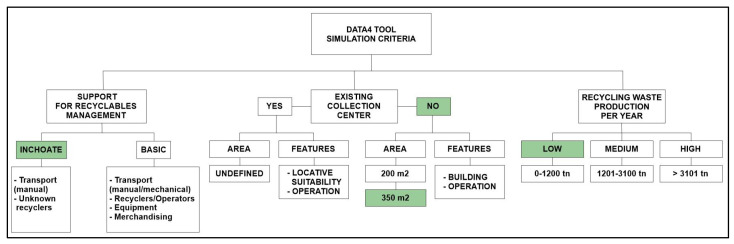
Attributes and variables included in the DATA4 tool for the case study.

Figure 8 shows the attributes and variables included in the DATA4 tool for the case study.

Guateque is a low-income municipality included under the sixth category in the Colombian classification. Some of its main characteristics are: (i) low-income granted by the national government; (ii) a low production of recyclable waste; (iii) absence of good practices for the segregation and/or use of waste and other elements considered for the model. These properties imply that the simulation should be developed in Scenario 1 of DATA4 (construction and operation of a storage center or waste treatment infrastructure).

### 3.2. Case Study: Component (G)

The municipality of Guateque has developed a Comprehensive Solid Waste Management Plan (CSWMP) for the period 2017–2028 that considers a 50% waste collection coverage on a weekly collection frequency [65]. The collected waste is transported and disposed of in the Pirgua landfill, which is located 92 km from the municipality. The monthly average of waste production is 264 tons. It does not have a scheme for the development of use of the recyclable material, there is no census of the recycling population and there is no installed infrastructure for the development of the activity.

A program focused on segregation at the source has been proposed, considering an efficiency equal to 80%. Some progress in composting activities and the generation of organic fertilizers that are distributed in agricultural projects with some economic incentives has also been considered [65].

Table 9 shows the composition of recyclable waste in the municipality of Guateque. Total annual recyclable waste production is equal to 63.60 t/year.

Although the quantities of production are low, the proximity of the municipality to other populated centers in the state with greater possibilities for the commercialization of recyclables (the regional management model could be successful) promotes the practice of segregation and the support of the model with it in waste management.

### 3.3. Case Study: Component (I)

DATA4 uses data provided by the municipal cleaning service company of the municipality of Guateque. Regarding the assessment of the collection service, it was established that the annual collection cost is EUR 13,505.77 and the reduction projection value to a 10-year horizon is EUR 2341.06, which means an important economic and environmental benefit by reducing expenses and considering improvements in reusable waste management.

**Figure 9:**
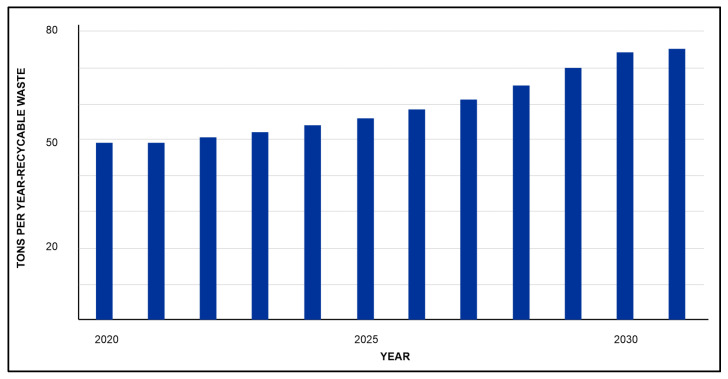
Projection of the recycling rate in Guateque (2020–2031).

Additionally, based on the data reported by the municipality, a projection of the collection of recyclable waste for a period of 10 years from 2020 was made. This projection considered that the recycling rate would progressively increase from 1% to 10%, as shown in Figure 9.

**Figure 10:**
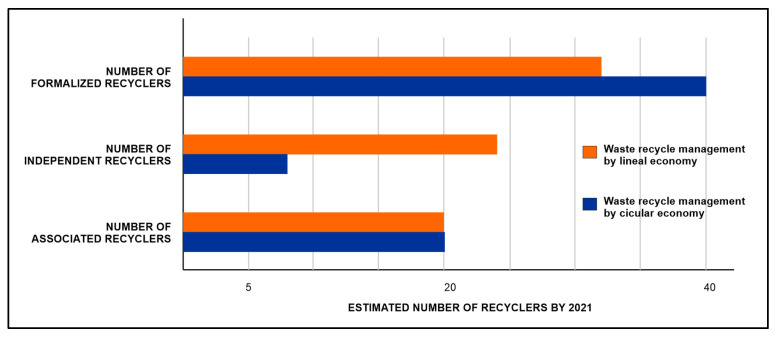
Estimated number of recyclers by type in the Guateque municipality.

In addition, a comparison between recyclable waste management through a linear economy model and a circular economy model was made. The analysis included the number of recyclers by type of service provision, distinguishing whether they are ex officio, whether they are formalized or whether they belong to an association. The results show that the number of formalized recyclers will gradually increase, decreasing those by trade and maintaining those belonging to associations, as illustrated in Figure 10. These results show that the circular economy model improves the technical capacity and administrative management of the cleaning and recycling activities, gradually legalizing and developing the service provided by recyclers.

Additionally, in the comparison between these two economic models on recyclable waste management, considering the recycler ratio by selective route recommended by Corredor in its environmental guide for waste management [66], the selective routes were projected to 2031 as the year in which the selective waste collection routes of recyclable waste should be higher in the circular model compared to the linear model. This explains how the circular model proposes an increase in formalized recyclers in charge of these selective routes. While for a circular economy approach the estimated number of routes is 12, for a linear economy approach this number decreases to only 3.

### 3.4. Case Study: Component (C)

The classification of collection centers in DATA4 is given by the ranges established by the Ministry of Housing, City and Territory, and the Ministry of Environment and Sustainable Development of Colombia [67]. Considering the information shown in Table 4 and following the attributes and variable classifications shown in Figure 8 and Table 8, Table 10 shows the configuration of the simulated collection center needing to be built for the case study.

### 3.5. Case Study: Component (T)

**Figure 11:**
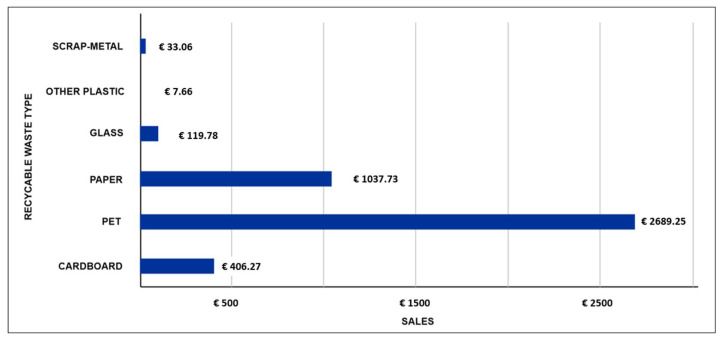
Results of the financial dashboard in DATA4: Projected sales by type of recyclable waste with an annual recycling rate from 1% to 10%.

Figure 11 shows the results of the economic valuation considering a gradual increase in the annual recycling rate of the Guateque municipality from 1% to 10%. Based on this information and the projection of the collected recyclable waste shown in Figure 9, sales were obtained by type of recyclable waste projected to 2031. These results are the product of the prices projected in 2031 of the usable materials mentioned in the Gómez-Franco methodology [68]. In this projection, the types of recyclable waste with the highest economic value are PET and paper, these being most generated according to Table 5.

Starting from results shown by Component (T), one general objective is to consolidate the union of external recyclable waste managers in low-income municipalities. This union has already been achieved at the national level in Colombia with organic and hazardous waste managers. In the organic waste case, some organizations have already created and consolidated alliances with large companies to manage the waste obtained from different production processes. One example is IIA-Engineering, Research and Environment, through technologies such as composting, reuse and treatment of used vegetable oils and waste compaction. They have succeeded in introducing or applying circular economy strategies to prevent these wastes from being disposed of in sanitary landfills [50].

### 3.6. Model Results and Analysis of Financial Viability

Cash flow analysis considered the costs of the collection, transportation and final disposal components, including the maintenance of the automotive fleet and the operational and administrative payroll. During the construction of the collection center, the sum of the costs of the workers’ salaries for 17 months (estimated construction time) were assumed. The 17 months correspond to the average construction period that a civil work of the dimensions considered for a collection center can take, such as the ones defined in the simulation with the model based on [69]. Costs of preliminary works, foundations, structuring and complementary works such as hydraulic installations were also considered. However, land purchase cost was not included. The budget base of the project was taken as established by García-Batista et al. [47], with a national inflation rate of 0.59% established by the National Administrative Department of Statistics of Colombia (DANE) for May 2021. The projection was made for the period 2021–2031 (10 years) taking as a reference studies carried out by the Ministry of Housing, City and Territory [70].

**Figure 12:**
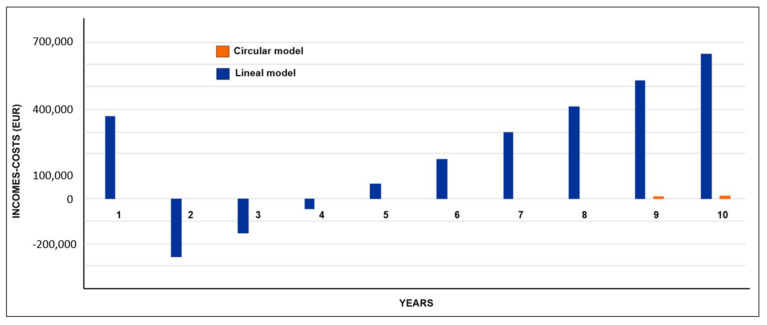
Results of the financial dashboard in DATA4: Cash flow projection (income–expenses) for the linear economy model and the circular economy simulation scenario.

**Figure 13:**
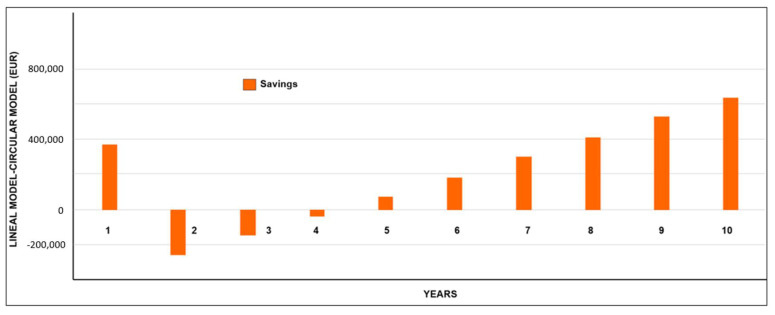
Results of the financial dashboard in DATA4: Cost reduction for collection, transport and final disposal of usable waste implemented in the simulation scenario (circular economy model).

Cash flow was obtained for the waste management models based on linear economy and circular economy. The results are compared in the financial control dashboard (Figure 12), indicating that in the first year incomes were higher than expenses. However, this effect was only due to the allocation of EUR 1,200,000 in governmental resources as a source of financing. In the same way, the cost reduction for the collection, transport and final disposal of recyclable waste was estimated by considering the difference between the cash flow of the circular economy model and that of the linear economy model, as shown in Figure 13.

Following the aforementioned cash flows, financial indicators are shown in Table 11. The results demonstrate that the application of the linear economy model is more viable in economic terms, due to the fact that it presents higher internal return rate (IRR) and cost benefit ratio (CBR) values. However, when comparing the results of the other indicators such as net present value (NPV) or the internal rate of opportunity (IRO), more favorable values were obtained as a result of the higher cash flow of the scenario of construction and operation of a collection center. Despite the above, the implementation of the latter is less economically profitable, because the IRR and the CBR are criteria with greater economic weighting than the NPV and the return period, according to the circular economy research conducted by Prieto-Sandoval et al. [56].

## 4. Discussion

For the circular economy model to be considered profitable and of interest to stakeholders, it is necessary to evaluate financial performance and ensure profitability. If the model also includes relevant effects on the environment, there is uncertainty as to whether “it is worth being ecological” or whether “to be ecological is not worthy”. In some models the costs of materials and energy are reduced because they do not induce significant expenses. However, there are other models in which the low cost of materials can be both their greatest advantage and their greatest obstacle [49].

The optimization of the circular economy model in terms of generating new commercial opportunities should consider that product reuse, re-manufacturing and reconditioning usually require fewer resources and energy than conventional recycling of materials, since these materials often remain in stock and do not generate any kind of profitability [36].

Using numerical tools such as M-GRCT to design circular economy models in recyclable waste management systems allows for reducing the materials that remain in stock by making intensive use of resources while minimizing environmental impacts derived from the exploitation and overproduction of resources. In addition, the implementation of circular economy models allows for promoting new business opportunities, adding value to production chains through innovation and the emergence of new business models that bring not only economic but social and environmental benefits [37].

Indeed, the inclusion of circular economy models entails a paradigm shift at all levels, starting with the change of public policies to gradually adopt the concept of circular economy at the legislative level to the detriment of the persistence of linearity in relation to the current economy. In other words, products become almost deterministically waste [38], particularly in the case of solid waste, followed by the population, who with the change in habits and improvements in their manner of consumption generate solutions; the same is true for the companies, which must generate environmental and financial sustainability, as well as for the academy, whose research contributes great knowledge and ideas for better inclusion [38].

Indeed, the inclusion of circular economy models in the field of waste management entails a paradigm shift. This change must be based on new public policies that gradually adopt the concept of circular economy, avoiding the linearity of the current economy in which products become almost deterministically waste [38]. All of this is particularly true in the case of solid waste management systems. Circular economy approaches must also change and improve the consumption habits of the population. Companies must also implement circular models that ensure environmental and financial sustainability. Finally, the contribution of academia, based on research results, should provide knowledge and ideas for a better inclusion of models based on circular economy [38].

Therefore, it is essential that the administration assume a relevant role in the implementation of a legislative and administrative framework that allows for promoting the development of strategies based on recycling and recovery of materials from the waste that is generated daily. All this must, in addition, guarantee the generation of economic benefits for companies while providing solutions to municipalities that apply these mechanisms to the management of domestic and industrial solid waste [71].

The development of models such as M-GCRT and numerical tools such as DATA4 represents a challenge in Colombia due to the lack of information on the provision of public cleaning services in low-income municipalities. The main advantage of integrating the circular economy model in a tool like DATA4 is its easy-to-use interface that does not require any license to implement it. As drawbacks we can point out that the economic valuation was carried out in a standardized way considering that the land where the waste collection center is to be built has low adaptation costs. As for any numerical model, prior calibration is needed, which is evidenced in the classification of simulation scenarios in DATA4.

During the execution of the DATA4 tool, with the information from the Guateque municipality, the economic viability was verified for both the linear (current) and circular solid waste management models, considering the positive NPV values obtained from the simulations. However, the IRR of the scenario of construction and operation of a waste collection center or infrastructure is lower than the IRR in the model based on linear economy, indicating that the project is not profitable. This could be explained because the income from the economic valuation does not include costs such as the rate adjustment for waste recovery approved by the Drinking Water and Basic Sanitation Regulation Commission (CRA) or benefits such as job creation, boosting the local economy and market strategies based on recyclable waste whose final destination is a sanitary landfill. In the same way, among other positive environmental impacts, the application of these models in low-income municipalities would induce a reduction in the emission of gases such as CO_2_. These last environmental benefits will be estimated by economic evaluation of impacts in a future version of DATA4 [72].

## 5. Conclusions

In low-income municipalities, deficiencies in local development and commerce, as well as inadequate traditional practices for waste management, were identified. Local governments of these municipalities must promote the reduction of the environmental impacts associated with such practices (e.g., burning) by implementing waste management models.

Waste management systems based on linear economy models favor some elements of the management chain, but they perpetuate the problems associated with final disposal sites and can even induce financial problems that can be strategically avoided by circular models. Waste management systems based on circular economy models require political will and efficient administrative operation together with active community participation.

This paper introduces M-GRCT, a new waste management model based on circular economy that is applicable to improving waste management in low-income municipalities with unfavorable socio-economic conditions. The components of the model suggest the coordination of the stakeholders involved to increase the separation of recyclable waste in the source. The model does not include the management of organic waste.

M-GRCT considers as important the participation of recyclers and the promotion of better marketing channels between them, the productive sector and cleaning services. These activities strengthen the waste management operations and promote municipal development.

The M-GRCT model was used successfully to analyze the management of recyclable waste in the municipality of Guateque (Colombia). The results show that the implementation of a model based on the circular economy would induce a greater increase in income and in the IRR than does the linear model. To perform a correct and complete interpretation of the model’s results, it would also be necessary to consider that the implementation of a model based on the circular economy induces a reduction in environmental impacts in a very important way. The economic evaluation of these impacts has not yet been incorporated into the model. Some of these positive impacts are the reduction of leachates and greenhouse gases, the reduction in soil degradation, the generation of jobs for local communities, the boost to the local and regional economy and the social improvements for the recycler’s associations. Future versions of M-GRCT and DATA4 will incorporate the evaluation of these positive environmental impacts.

## Figures and Tables

**Table 1:** Full parameter list: DATA4.

Block 1. Main Menu		Block 2. Information Registry		Block 3. Database and Scenario Classification	
Variable	Unit	Variable	Unit	Variable	Unit
1. Recycling waste first input of data	--	1. Total incomes and expenses of the waste collection (linear model)	Euros	1. Presence of waste valorization station(s)	Yes/No
2. Implementation of scenario monitoring	--	2. Presence of waste classification station(s)	Yes/No	2. Recyclable waste collection type (small-medium-large)	t/year
		3. Annual generation of recyclable waste	t/year		
		4. Waste infrastructure classified according to surface area	Yes/No		
**Block 4. Economic evaluation results**		**Block 5. Financial control dashboard**		**Block 6. Implementation of scenario monitoring dashboard**	
**Variable**	**Unit**	**Variable**	**Unit**	**Variable**	**Unit**
1. Scenario	--	1. Recycling rate	%	1. Total number of recyclers	#
2. Cost structure of the cleaning service provider	Euros	2. Total income and expenses of waste collection system (linear/circular model)	Euros	2. Number of associated recyclers	#
3. Annual operating budget	Euros	3. Cost reduction due to the implementation of scenarios.	Euros	3. Number of formalized recyclers	#
4. Building cost of new infrastructures	Euros	4. Internal return rate (IRR) of the waste management system (linear/circular model)	%	4. Number of selective collection routes	
5. Rehabilitation costs of infrastructures	Euros	5. Investment return period	years		

**Table 2:** Environmental context processing into the macro-type array.

Feature	Variable	Unit	Mathematical Process
1. Reduction of carbon footprint by transportation	- Operating capacity (a)	t	Capacity 1 = Compactor capacity (m^3^)·Density of compacted waste (t/m^3^)
			Capacity 2 = Vehicle capacity (m^3^)·Density of uncompact waste (t/m^3^)
			a = ∑capacity 1 + ∑capacity 2
	- Annual projection of the weight of recyclable waste disposed of in landfills (b)	t/year	Percentage increase (2%/year) after implementing the circular model
	- Rejection percentage (c)	%	=20% of the total reported in the main menu
	- Weight of recyclable waste processed (d)	t/year	=b·c
	- Annual frequency to the landfill (e)	#/year	=Data registered in the main menu
	- Distance to the landfill (f)	km	=Data registered in the main menu
	- Distance to the transformation place (g)	km	=5 km by default
	- Traveled distance per year (h)	km	=d·(e−f)
	- GHG-IPCC emission factor (i)	KgCO_2_e/km	=0.68653 KgCO_2_e/km by default assuming Heavy Goods Vehicles (IPCC)
	- Reduction of the carbon footprint by transportation (j)	KgCO_2_e	=h·i
2. Reduction of carbon footprint due to the generation of leachate	- Annual projection of the weight of recyclable waste disposed of in landfills	t/year	=b
	- GHG-IDEAM emission factor (k)	KgCO_2_e/t	=0.022 KgCO_2_e/t by default assuming IDEAM criteria
	- Reduction of the carbon footprint due to the generation of leachate (l)	KgCO_2_e	=b·k
3. Reduction of carbon footprint by gas generation	- Methane gas emissions in landfill (m)	t/year	EPA LandGEM fill model:$Q_{CH4}=\overset{n}{\underset{i=1}{\sum }}\cdot \overset{1}{\underset{j=0.1}{\sum }}KLo\cdot \left(\frac{Mi}{10}\right)\cdot e^{-k\cdot t_{ij}}$
	- Ratio of recyclable municipal waste/total waste in landfill (n)	t	=b/data reported in the SUI and stored in DATA4
	- Municipal methane gas emissions (o)	t/year	=m·n
	- GHG-IPCC emission factor (p)	KgCO_2_e/t	=21 KgCO_2_e/t by default assuming IPCC criteria
	- Carbon footprint due to gas generation (q)	KgCO_2_e	=o·p
4. Promotion of an increase in the number of associated recyclers	- Weight of recyclable waste processed	t/year	=d
	- Ratio of professional recyclers/t of recyclable waste (r)	#/t	=Data registered in the main menu/d
	- Relation associated recyclers/t of recyclable waste (s)	#/t	=Data registered in the main menu/d
	- Number of professional or informal recyclers (t)	#	=Data registered in the main menu
	- Number of recyclers associations (u)	#	=Data registered in the main menu
5. Promotion of an increase in the number of selective routes	- Weight of recyclable waste processed	t/year	=d
	- Number of selective routes (v)	#	=Data registered in the main menu
	- Relation selective routes/t of recyclable waste	#/t	=Data registered in the main menu/d

Note: Features 1 and 2 correspond to recyclable waste that will not reach the landfill. GHG: Greenhouse gases. IPCC: Intergovernmental Panel on Climate Change. IDEAM: Colombian Institute of Hydrology, Meteorology and Environmental Studies. SUI: Colombian Unified information system for home public services.

**Table 3:** Financial context processing into the macro-type array.

Feature	Variable	Mathematical Process/Function
1. Linear Model Egress Structure	- Revenue	=monthly average recyclable waste production + monthly municipality budge
	- Expenditure	=operational costs + expenses
2. M-GRCT Model Egress Structure	- Revenue	=monthly average recyclable waste production + monthly municipality budget + tariff adjustment + recovery of usable waste
	- Expenditure	Simulation scenario 1 = operational costs + expenses + investment in infrastructure + construction operational costs
		Simulation scenario 2 = operational costs + expenses + investment in locative adjustments + construction operational costs
	- Economic rescue	Corresponds to the value allocated by the national participation fund for the operation of public cleaning service companies, because they have a low budget and do not have the necessary resources for its operation.
3. Funding requirements	- Repayment	Payment (Interest rate; term; capital)
	- Interest	Capital·annual effective rate
4. Financial ratios	- Net Present Value (NPV)	Rate; Periods; Total cash flow
	- Internal Rate of Return (IRR)	Periods; Total cash flow1; net cash flow year10
	- Internal Rate of Opportunity (IRO)	Assumed value of 10%
	- Cost Benefit ratio	=revenue-expenditure

Note: Features such as taxes and depreciation were not considered due to limitations in obtaining the information.

**Table 4:** Characterization of recyclable waste DATA4.

Waste	Measurement Unit
Paperboard	t/year
PET	t/year
Paper	t/year
Glass	t/year
Other plastics	t/year
Scrap-metals	t/year

**Table 5:** Information record related to Component (R): DATA4.

Variable	Measurement Unit	Block
Recyclable waste collection per year	t/year	2
Number of independent recyclers	#/year	6
Number of associated recyclers	#/year	6
Number of formalized recyclers	#/year	6
Number of waste selected routes	#/year	6

**Table 6:** Information record related to Component (C): DATA4.

Collection Center Area			Recyclable Waste	Collector Type	Scenario
Existing Center m^2^	Non-Existent Center				
	200 m^2^	350 m^2^			
#	#	#	t/year	Small/medium/high	1 or 2

**Table 7:** Information related to Component (T)-DATA4.

Variable	Measurement Unit	Block
Recyclable rate	%	5
Sales by recycle waste type Paperboard PET Paper Glass Other plastics Scrap-materials	Euros	5

**Table 8:** Registry and general information of the Guateque municipality.

Sub-block	Parameter	Value	Measurement Unit
General information	Department	Boyacá	--
	Municipality	Guateque	--
	Economy category	6	#
	Waste collecting company	Empresa de servicios públicos y aseo de Guateque	--
Recycle information	Recyclable waste collected per year	49	t/year
	Existing collection center	NO	YES/NO
	Registered recyclers	-	#
	Simulation collection center area *	350	m^2^

* For the Colombian classification of municipalities, a constructed area of 200 m^2^ is simulated for the fifth category and 350 m^2^ for the sixth category. Both categories are considered low-income.

**Table 9:** Composition of recyclable waste: Guateque.

Waste	Production (t/year)	Percentage (%)
Paperboard	11.13	17.50%
PET	29.57	46.49%
Paper	13.14	20.66%
Glass	9.13	14.36%
Other plastics	0.12	0.19%
Scrap-metals	0.51	0.80%

**Table 10:** Data related to Component (C): DATA4.

Collection Center Area			Recyclable Waste (t/year)	Collector Type	Scenario
Existing Center m^2^	Non-Existent Center				
	200 m^2^	350 m^2^			
0	0	1	49	Small	1

**Table 11:** Financial viability evaluated at 10 years (2021–2031) of the simulated model and the linear economy model.

Financial Indicator	Waste Management Model Based on Linear Economy	Waste Management Model Based on Circular Economy
VPN	EUR 12,364.88	EUR 649,154.69
IRR	1.28%	1.14%
IRO	10%	10%
CBR	17%	9%
Payback period	8.3 years	5.2 years

## Data Availability

Data used in this research were obtained from different sources: (i.) The Solid Waste Management Plan (SWMP) of Guateque; (ii.) The 2018 Solid Waste Final Disposal National Report of Colombia; (iii.) The 2018 National Waste Reuse Report of Colombia (restrictions apply to the availability of these data); (iv.) the National Planning Department (NDP); (v.) the Public services Superintendence of Colombia’s usable report from 2019 on industrial waste; and (vi.) the Statistics National Department (SNP). Data are available from the authors with the permission of the corresponding local or national Colombian Authority.
